# Heterologous expression of the hypovirus CHV1-EP713 full-length cDNA in *Botrytis cinerea*: transformation with *Agrobacterium tumefaciens* and evaluation of changes in the fungal phenotype

**DOI:** 10.1186/s40659-025-00645-y

**Published:** 2025-10-08

**Authors:** Luis Cottet, Grace Armijo-Godoy, Antonio Castillo

**Affiliations:** 1 Laboratorio de Control Biológico y Nanotecnología, Departamento de Biología, Facultad de Química y Biología, Universidad de Santiago de Chile (USACH), Av. L. B. O’Higgins 3363, 9170022 Estación Central, Santiago Chile; 2 Escuela de Agronomía, Facultad de Ciencias, Ingeniería y Tecnología, Universidad Mayor, 4801043 Temuco, Chile; 3 Present Address: Centro de Genómica Nutricional Agroacuícola, CGNA, Temuco, Chile

**Keywords:** *Botrytis cinerea*, *Cryphonectria parasitica*, *Agrobacterium tumefaciens*, Hypovirus, Mycovirus, Hypovirulence, Transformation

## Abstract

**Background:**

*Botrytis cinerea* is a phytopathogenic fungus responsible for gray mold disease in a wide range of hosts, including ornamentals, vegetables, and fruit-bearing plants. Similarly, *Cryphonectria parasitica* infects the American chestnut, causing a lethal condition known as chestnut blight. From this species, the CHV1-EP713 virus, classified as a hypovirus due to its ability to reduce fungal virulence, has been isolated and characterized. Building on this knowledge, we aimed to express the full-length cDNA of CHV1-EP713 in *B. cinerea* to asess whether its expression could alter the fungal phenotype.

**Results:**

To achieve the expression of the hypovirus cDNA in *B. cinerea*, the pXH9 vector encoding the CHV1-EP713 cDNA and the p18 plasmid containing the *Agrobacterium tumefaciens* Ti plasmid T-DNA region were fused to generate the p18-XH9 construct. Transformation of the virulent *B. cinerea* strain CCg55L with *A. tumefaciens* carrying this construct yielded hygromycin B-resistant transformants. Nucleic acid analysis revealed a ~ 13-kbp double-stranded RNA, consistent with a replicative intermediate of the viral genome. PCR and RT-PCR confirmed integration and expression of the viral cDNA, supporting the establishment of a productive mycoviral infection. Phenotypically, transformants showed reduced radial growth and sporulation compared to the parental strain. Moreover, grapevine leaf infection assays revealed significantly reduced tissue damage and distinct oxidative responses, indicating a reduction in virulence.

**Conclusion:**

Together, these results demonstrate that transformation of a virulent *B. cinerea* strain with CHV1-EP713 cDNA can lead to phenotypic changes consistent with hypovirulence. The observed alterations in growth, sporulation, and pathogenicity are likely linked to viral expression and/or replication, highlighting the potential of hypoviruses as biological control agents against phytopathogenic fungi.

**Supplementary Information:**

The online version contains supplementary material available at 10.1186/s40659-025-00645-y.

## Introduction

Mycoviruses have been isolated and characterized in numerous filamentous fungi and yeast species [1]. Most characterized fungal viruses possess either double-stranded RNA (dsRNA) or single-stranded RNA (ssRNA) genomes. Viruses with linear positive-sense ssRNA genomes are classified into the families *Alphaflexiviridae*, *Barnaviridae*, *Deltaflexiviridae*, *Endornaviridae*, *Gammaflexiviridae*, *Hypoviridae*, *Narnaviridae*, *Fusariviridae*, *Mitoviridae*, *Hadakaviridae*, and *Yadokariviridae*, as well as the reverse-transcribing (RT) families *Metaviridae* and *Pseudoviridae* [2–6]. As no extracellular infection cycle has been described for fungal viruses, their transmission occurs exclusively through intracellular routes [1, 3]. Horizontal transmission mainly takes place via hyphal anastomosis between compatible individuals, whereas vertical transmission occurs through asexual spores produced during mitotic division of the infected fungus [3].

Most mycovirus infections are cryptic or latent, producing no noticeable phenotype in the host fungus and making it challenging to distinguish infected from virus-free isolates [1]. Nevertheless, some viral infections induce characteristic symptoms. For example, infection by the totivirus HvV190S in *Helminthosporium victoriae* results in reduced colony size, lysis of aerial mycelium, and low sporulation rates [7]. Another example is the killer phenotype in *Saccharomyces cerevisiae*, where strains harboring the totivirus ScV-LA and a satellite virus (ScV-M1, ScV-M2, or ScV-M28) produce a toxin lethal to virus-free *S. cerevisiae* strains [8–10]. A third example is the hypovirulence observed in *Cryphonectria parasitica*, where infection with the CHV1-EP713 hypovirus results in reduced virulence, low sporulation, loss of pigmentation, and diminished damage to the host plant [11–13].

CHV1-EP713, the first hypovirus characterized from the chestnut blight fungus *C. parasitica* [12], has a linear, positive-sense ssRNA genome of 12,734 nucleotides (nt) containing two open reading frames (ORF-A and ORF-B) that encode four proteins [14, 15]. ORF-A encodes a 69 kDa polyprotein (p69) that undergoes autoproteolytic cleavage to generate a papain-like protease (p29) and a highly basic protein (p40), derived from its N- and C-termini, respectively. ORF-B encodes a polyprotein that, upon cleavage, produces p48 and an RNA-dependent RNA polymerase [12, 16]. Proteins p29, p40, and p48 are involved in regulating and reducing fungal virulence [12]. Reverse genetics experiments, in which modified CHV1-EP713 genomes were expressed in virus-free *C. parasitica*, revealed that p29 suppresses RNA silencing, decreases pigmentation, asexual sporulation, and extracellular laccase activity, but is not directly associated with viral replication or the degree of hypovirulence [17–19]. Protein p40 is also linked to pigmentation loss, reduced sporulation, and increased viral RNA accumulation [20]. Meanwhile, p48 is associated with initiating viral RNA accumulation, influencing canker size in infected trees, and, to a lesser extent, suppressing pigmentation and conidiation [12, 21]. Collectively, these proteins contribute to varying degrees, to the hypovirulent phenotype of CHV1-EP713–infected fungi.

In this context, since no simple method exists to purify the hypovirus or its genome, reverse genetics systems for CHV1-EP713 have been developed to investigate its infection mechanisms and contribution to hypovirulence in *C. parasitica* [12]. Two infection strategies are commonly used. The first involves the fungal transformation and chromosomal integration of the pXH9 plasmid, which carries a cDNA copy of the hypoviral genome under the control of the *C. parasitica* glyceraldehyde-3-phosphate dehydrogenase promoter and terminator [14, 22]. The second strategy relies on transfection with synthetic mRNAs generated in vitro from the pLDST vector [12, 23]. Both methods have confirmed that CHV1-EP713 infection confers hypovirulence in *C. parasitica* [22, 23] and have also been used to establish infection in *C. radicalis*, *C. cubensis*, *Endothia gyrosa*, and in more distantly related fungi such as *Valsa ceratosperma* and *Phomopsis* G-type [23, 24]. In all cases, the hypovirus replicated, produced dsRNA, and induced phenotypic changes, demonstrating that its host range extends beyond a single species or genus.

The methods mentioned above require fungal transformation. The most employed techniques for this purpose are spheroplast fusion, biolistics, and *Agrobacterium tumefaciens*–mediated transformation, the latter resembling plant transformation methods [22, 24–26]. *A. tumefaciens* is a phytopathogenic bacterium that causes crown gall disease by transferring a segment of its Ti plasmid, termed T-DNA, into the plant genome, where its expression triggers uncontrolled cell proliferation [26]. This transfer is mediated by virulence (*vir*) gene products encoded on the Ti plasmid [27]. The T-DNA region, flanked by right (RB) and left (LB) border sequences, can be engineered to carry foreign genes instead of the native tumor-inducing genes [27]*.* For fungal transformation, modified T-DNA constructs have been designed to express various genes, including reporter and antibiotic resistance markers [26]. Such constructs have been successfully used to transform fungi, including *Botrytis cinerea*, *Eupenicillium parvum*, *Aspergillus terreus*, *Aspergillus niger*, *Neurospora crassa*, *Fusarium venenatum*, and *Venturia inaequalis* [28–31].

*Botrytis cinerea* Pers. [teleomorph *Botryotinia fuckeliana* (de Bary) Whetzel] is a necrotrophic fungus infecting over 1,000 plant species [32]. It causes grey mold disease, which affects numerous ornamental, vegetable, and fruit crops of high economic value [33]. Mycoviruses have been identified in several *B. cinerea* strains, and research has focused on characterizing viral particles and genomes, as well as on determining the relationship between viral infection and fungal phenotypes [34].

Given that *B. cinerea* and *C. parasitica* are both filamentous ascomycetes, we hypothesized that they may share similar molecular mechanisms. Moreover, since *B. cinerea* can be efficiently transformed using *A. tumefaciens* [30], we propose that transforming a virus-free, virulent strain of *B. cinerea* with the pXH9 plasmid via *A. tumefaciens*–mediated transformation, will lead to expression of the CHV1-EP713 genome, thereby inducing phenotypic changes in the transformed fungus.

## Materials and methods

### Strains and culture conditions

*Botrytis cinerea* CCg55L was isolated from Thompson Seedless grape berries (*Vitis vinifera*). The fungus was grown on MYA medium (1.5% malt extract, 0.75% yeast extract, and 1.5% agar) for 5 to 7 days at 20 °C in darkness. *Escherichia coli* DH5α was obtained from Invitrogen (Carlsbad, California, USA) and was cultured on LB medium (1.0% peptone, 0.5% yeast extract, 1.0% NaCl, and 1.5% agar) for 24 h at 37 °C. *Agrobacterium tumefaciens* LBA1126 was kindly donated by Dr. Christophe Bruel (Laboratoire de Biologie Cellulaire Fongique, Université Claude Bernard Lyon, France) and was grown on LB medium for two days at 28 °C.

### Plasmid construction

**Fig. 1:**
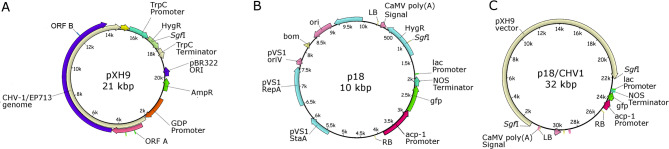
Schematic representation of the plasmids used and generated in this study, showing the relevant genes for cloning. **A** The pXH9 vector contains the full-length cDNA of the hypovirus CHV1-EP713 and the hygromycin B phosphotransferase gene (*hph*). **B** The p18 plasmid contains the T-DNA region flanked by the left and right borders (LB and RB), the *hph* gene, and the green fluorescent protein (*gfp*) gene. **C** The p18-XH9 vector was constructed by fusing the pXH9 and p18 plasmids. The T-DNA region contains the elements that will be transferred to the fungus. The position of the *Sgf*I restriction site used for plasmid fusion is indicated in all three plasmids

Since the p18 plasmid of *A. tumefaciens* lacks a multicloning site, the CHV1-EP713 hypovirus cDNA contained in plasmid pXH9 was cloned by fusing both vectors. Both plasmids contain an *Sgf*I restriction site within the gene encoding hygromycin B phosphotransferase (Fig. 1). Therefore, they were digested with *Sgf*I, linearized, and subsequently ligated to generate the p18-XH9 plasmid. This plasmid contains the CHV1-EP713 hypovirus cDNA inserted between the right (RB) and left (LB) borders of the *A. tumefaciens* plasmid, preserving the integrity of the hygromycin B resistance gene. The resulting p18-XH9 plasmid was maintained in *E. coli*, purified, and used to transform *A. tumefaciens*.

### Botrytis cinerea transformation

*A. tumefaciens* was transformed as previously described [30]. Bacteria carrying the p18-XH9 plasmid were maintained in LB medium supplemented with ampicillin (100 μg/mL), kanamycin (100 μg/mL), and spectinomycin (250 μg/mL) (LB-SAK medium). Mycelia and conidia of *B. cinerea* CCg55L were purified as described [25]. Fungal mycelium was cultured in two Erlenmeyer flasks with 50 mL of MY medium for 15 days at 20 °C. The mycelium (8–10 g) was macerated in 10 mL of distilled water using a homogenizer, then filtered through sterile gauze and centrifuged at 8000 × g for 10 min. The pellet was resuspended in 1 mL of distilled water. For conidia production, the fungus was cultured in flasks with 7.5 g/L malt extract and 15 g/L agar for 15 days. Sterile water was added and shaken vigorously for 30 min to recover spores, which were then filtered and centrifuged as described above.

*B. cinerea* was transformed with *A. tumefaciens* following the method in [30], with slight modifications. *A. tumefaciens* was grown in LB-SAK medium for 12 h at 28 °C. The culture was then diluted in induction medium (10 mM glucose, 0.5% glycerol, and 200 μM acetosyringone in 10 mM phosphate buffer pH 7.0, 2.5 mM NaCl, 2 mM MgSO_4_, 0.45 mM CaCl_2_, 9 μM FeSO_4_, 4 mM (NH_4_)_2_SO_4_) to an OD600 of 0.15 and incubated under the same conditions until reaching an OD600 of 0.4–0.5. For transformation, 100 μL of bacteria and 100 μL of a 1 × 10⁷ conidia/mL suspension, or 100 μL of macerated mycelium, were mixed and inoculated onto solid induction medium over nitrocellulose paper disks. The plates were incubated in the dark at 22 °C for two days. To select *B. cinerea* transformants, the nitrocellulose membranes were transferred to solid MY medium supplemented with 200 μg/mL hygromycin B and 200 μM cefotaxime and incubated for 15 days at 20 °C (Supplementary material, Fig. S1).

### Molecular analysis of T-DNA integration

Genomic DNA was purified from transformed *B. cinerea* mycelium and used for thermal asymmetric interlaced PCR (TAIL-PCR). The fungal tissue was grown in Erlenmeyer flasks with MY medium for 15 days at 20 °C. Subsequently, the mycelium was collected with forceps, and excess culture medium was removed by pressing it between sterile paper towels. Two hundred milligrams of mycelium were macerated in 250 µL of STE 2X buffer containing 0.2% β-mercaptoethanol, followed by the addition of 250 µL of STE 2X buffer containing 2% SDS, and homogenized for 30 s using a vortex. Nucleic acids were extracted using equal volumes of phenol:chloroform:isoamyl alcohol (25:24:1). The mixture was vortexed for 30 s and centrifuged at 10,000 × g for 10 min. The aqueous phase (400 μL) was transferred to a fresh tube, mixed with 800 μL of cold ethanol, and incubated overnight at − 20 °C. Nucleic acids were pelleted by centrifugation at 12,000 × g for 15 min at 4 °C. The pellet was then dried and resuspended in 100 μL of distilled water. Total nucleic acids were digested with RNase A as described [35], extracted once with phenol:chloroform:isoamyl alcohol (25:24:1), and then with chloroform:isoamyl alcohol (24:1). Finally, genomic DNA was quantified using a UV–VIS spectrophotometer (Shimadzu) and visualized on a 0.7% agarose gel stained with GelRed (Biotium).

The genomic DNA region of *B. cinerea* where the T-DNA was inserted was determined by TAIL-PCR using the primers shown in Table 1. Genomic DNA (30 ng) from *B. cinerea* transformants was used as a template for the first TAIL-PCR. The reaction mixtures and conditions for the first, second, and third TAIL-PCR were as described in [36]. The products of the third TAIL-PCR were visualized by agarose gel electrophoresis and sequenced using the RB3 primer as described in [37].

**Table 1:** Primers used to amplify and sequence the genomic DNA flanking the T-DNA

Primer	Sequence (5′ → 3′)
RB1	GGCACTGGCCGTCGTTTTACAAC
RB2	AACGTCGTGACTGGGAAAACCCT
RB3	CCCTTCCCAACAGTTGCGCAG
AD1	WAGTGNAGWANCANAGA
AD2	TGWGNAGSANCASAGA
AD4	STTGNTASTNCTNTGC

### Detection of viral mRNA

RNA from the wild-type strain and transformant clones of *B. cinerea* was purified from total nucleic acids. Fungal mycelium was macerated as described above, and RNA was purified using the E.Z.N.A Total RNA Kit (Omega Bio-Tek). Viral mRNA was detected by RT-PCR using two primer pairs that amplify ~ 500-nt fragments from ORF-A and ORF-B. For ORF-A, primers ORFA930F (5′-CCTGTGCTCATCTCGGAACGGT-3′) and ORFA1493R (5′-TCGTATCTCGCCCAAAGGCGACGA-3′) were used, while ORFB9986F (5′-AGTCTACGGACTGGCGAACACGTT-3′) and ORFB10480R (5′-GGAATCTCCCAGTTATCGCGGAGG-3′) were employed for ORF-B. The AffinityScript cDNA Synthesis Kit (Agilent Technologies) was used for cDNA synthesis in a reaction containing 1 μg of total RNA, 0.5 μg of primer (ORFA1493R or ORFB10480R), and 7.7 μL of distilled water. The mixture was incubated at 65 °C for 5 min. Then, 2 μL of 10X buffer, 25 mM of each dNTP, 20 U of RNasin, and 1 μL of AffinityScript Multiple Temperature RT were added. The final reaction was incubated at 50 °C for 60 min. PCR was performed using both primer sets. As DNA templates and controls, the following were included: (i) the vector pXH9 as a positive control; (ii) total DNA from transformed strain to verify cDNA integration into the fungal genome; (iii) total RNA from transformed *B. cinerea* and total nucleic acids from *B. cinerea* CCg55L, both as negative controls; and (iv) the RT product to detect viral mRNA. PCR reactions (25 μL) contained 19.05 μL of water, 2.5 μL of 10X buffer, 25 mM of each dNTP, 1 μL of 10 mM of each primer, 50–100 ng of template, and 0.125 U of Paq500 DNA polymerase (Agilent). The amplification program consisted of an initial denaturation at 94 °C for 2 min, followed by 35 cycles of denaturation at 94 °C for 40 s, annealing at 55 °C for 40 s, elongation at 72 °C for 30 s, and a final elongation at 72 °C for 5 min. Amplification products were resolved by agarose gel electrophoresis as described above.

### DsRNA purification and analysis

The wild-type and transformant clones of *B. cinerea* were grown in 250-mL Erlenmeyer flasks containing 50 mL of MY medium for 15 days in darkness at 20 °C. Replicative intermediates of the hypovirus genome (double-stranded RNA, dsRNA) were purified from total fungal nucleic acids using CF-11 cellulose chromatography, as described [38]. Its double-stranded nature was validated by its resistance to DNase I, S1 nuclease, and RNase A as described in [39].

### Determination of growth and sporulation rates

Actively growing mycelia from wild-type and transformant strains of *B. cinerea* were inoculated at the center of Petri dishes containing solid MY and King’s B media (2% peptone, 0.15% K_2_HPO_4_, 0.15% MgSO_4_, 1% glycerol, and 1.5% agar) and incubated for five days in darkness at 20 °C. Radial growth was measured daily for five days from the center to the colony edge. Growth rates were expressed in millimeters per day (mm/day).

To determine sporulation rates, wild-type and transformant strains were grown in 250-mL Erlenmeyer flasks with 50 mL of MY and King’s B media for 14 days at 20 °C. Spores were purified as previously described and quantified using a Neubauer chamber (Marienfeld).

### Virulence bioassays and determination of reactive oxygen species (ROS) production on grapevine leaves

For the virulence assay, grapevine leaves were surface-sterilized by immersion in 0.05% sodium hypochlorite for 1 min, followed by three rinses with sterile distilled water. Five-millimeter mycelial plugs were excised from 2–3-day-old cultures grown on MYA medium and placed at the center of intact leaves. The lesion area was measured after four days of incubation in the dark at 20 °C. To assess ROS generation in response to fungal infection, grapevine leaves were inoculated with 10 µL of a 1 × 10^5^ conidia/mL suspension and incubated at 20 °C for 24 h under 12 h light/12 h dark cycles. Infected areas were excised and placed on a drop of 3,3′-diaminobenzidine (0.1% DAB in 6.7% DMSO) and vacuum-infiltrated for 7 min. Leaf discs were incubated for 3 h under light and then washed with 80% ethanol for 5 min at 60 °C. For fungal staining, leaf discs were clarified with 96% ethanol for 10 min, stained with trypan blue (0.025% trypan blue, 28.3% glycerol, and 30% lactic acid) for 30 min, and incubated in fixing solution (28.3% glycerol and 30% lactic acid) for 90 min at room temperature. Samples were analyzed using light microscopy (Olympus) at 40X magnification.

### Statistical analysis

All experiments were performed with four biological replicates. Analysis of variance (ANOVA) was performed among the tested conditions using R software. Tukey’s post hoc test was applied to determine significant differences among treatments at a *p*-value < 0.05.

## Results

### Transformation of Botrytis cinerea mediated by Agrobacterium tumefaciens

To construct the transformation vector containing the cDNA of hypovirus CHV1-EP713, the *A. tumefaciens* plasmid p18 was fused with pXH9 using the unique restriction site for the enzyme *Sgf*I, located within the hygromycin B phosphotransferase (*hph*) gene (for details, see Materials and Methods) (Fig. 1). This strategy generated two recombinant plasmids, p18-XH9-A2 and p18-XH9-K2, as independent clones to control for variation during vector construction and to ensure that the observed phenotypes were not the result of rare cloning artifacts. Both vectors were used to transform *B. cinerea* CCg55L, and the resulting transformants were designated CCg55L-A2 and CCg55L-K2.

### Genomic insertion site of T-DNA

**Fig. 2:**
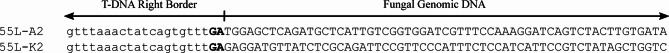
Sequences obtained by TAIL-PCR showing the flanking regions between the T-DNA and *B. cinerea* genomic DNA. Lowercase letters indicate T-DNA sequence; bold uppercase letters correspond to remnants of the right T-DNA border; non-bold uppercase letters represent fungal genomic DNA. The transformant strain names are indicated on the left

Thermal asymmetric interlaced PCR (TAIL-PCR) was used to identify the *B. cinerea* genomic regions where T-DNA was inserted. Sequencing of the PCR products revealed that T-DNA was inserted in different chromosomes: BCIN04 for CCg55L-A2 and BCIN09 for CCg55L-K2. BLAST analysis indicated that the insertion site in CCg55L-K2 is located in an intergenic region, while in CCg55L-A2 it disrupts a gene encoding the hypothetical protein BC1G_04546, whose function remains unknown (Fig. 2).

### Detection of viral mRNAs in B. cinerea transformants

**Fig. 3:**
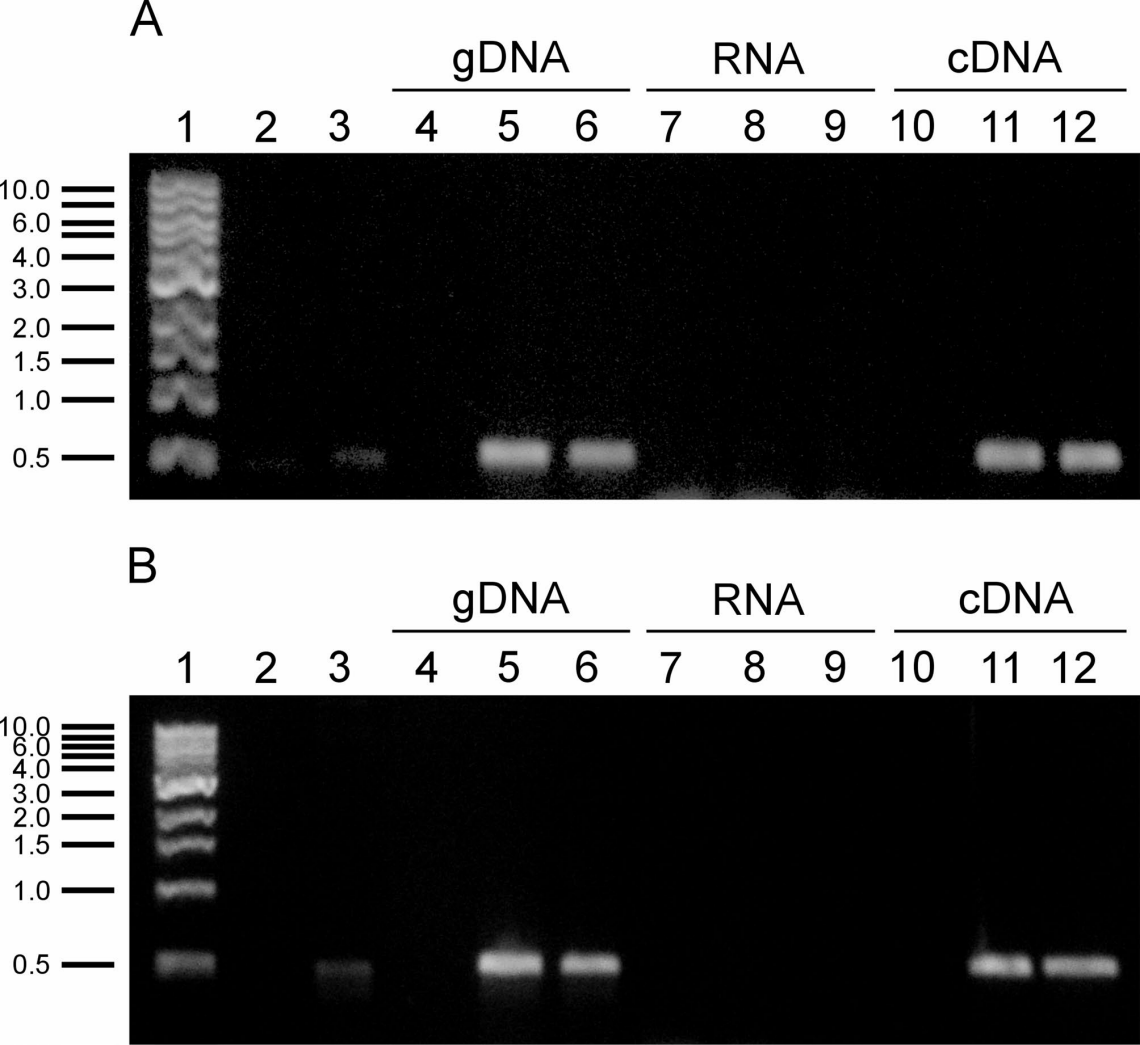
RT-PCR detection of CHV1-EP713 mRNAs in *B. cinerea* CCg55L-A2 and CCg55L-K2 strains. **A** Amplification using primers for ORF-A. **B** Amplification using primers for ORF-B. Samples correspond to genomic DNA (gDNA), total RNA (RNA), and reverse-transcribed RNA (cDNA). Lane 1, 1 kb DNA ladder; lane 2, PCR negative control; lane 3, pXH9 vector; lanes 4, 7, and 10, *B. cinerea* CCg55L; lanes 5, 8, and 11, *B. cinerea* CCg55L-A2; lanes 6, 9, and 12, *B. cinerea* CCg55L-K2. The numbers on the left correspond to molecular sizes in kilobase pairs (kbp)

To determine whether the CHV1-EP713 hypovirus cDNA or dsRNA was transcribed, viral mRNAs were detected by RT-PCR using two primer pairs targeting ~ 500-nt regions within ORF-A and ORF-B. Genomic DNA from the transformants was used as a positive control to detect integrated viral cDNA. *B. cinerea* CCg55L DNA and the pXH9 plasmid were used as negative and positive controls, respectively. As expected, ~ 500 bp fragments were amplified when either genomic DNA or the RT-PCR product was used as the template (Fig. 3). No amplification was observed when total RNA was used directly, indicating the absence of contaminating viral RNA or DNA. Notably, no products were amplified from CCg55L controls, confirming the primers specificity for the viral genome.

### Purification and characterization of dsRNA

**Fig. 4:**
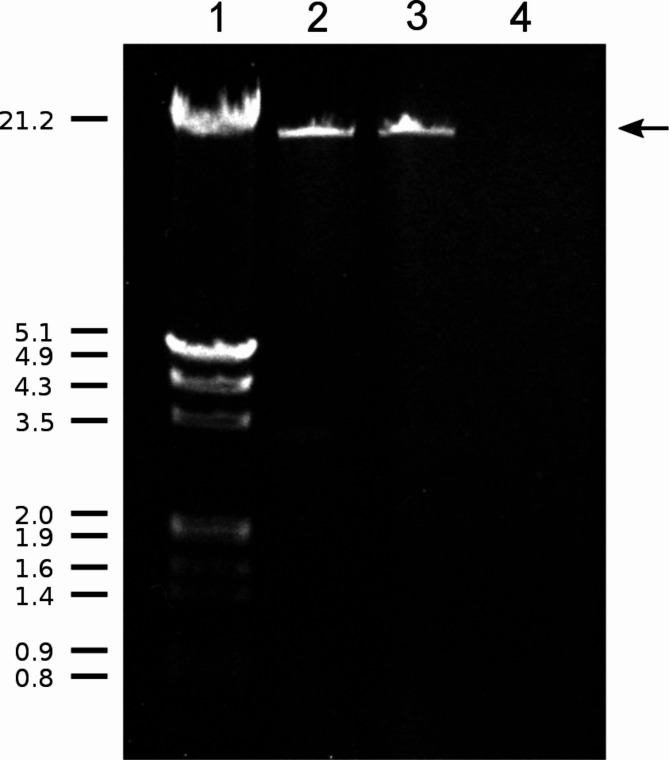
Agarose gel electrophoresis of dsRNA purified from *B. cinerea*. Lane 1, λ DNA digested with *Eco*RI and *Hin*dIII; Lane 2, CCg55L-A2 dsRNA; Lane 3, CCg55L-K2 dsRNA; Lane 4, CCg55L dsRNA. The arrow indicates the dsRNA corresponding to CHV1-EP713 replicative intermediates. The numbers on the left correspond to molecular sizes in kilobase pairs (kbp)

Expression of the hypovirus genome was further confirmed by purifying dsRNA using CF11-cellulose chromatography. A ~ 13-kbp dsRNA was isolated from both transformants (Fig. 4).

### Phenotypic changes in transformed strains

**Fig. 5:**
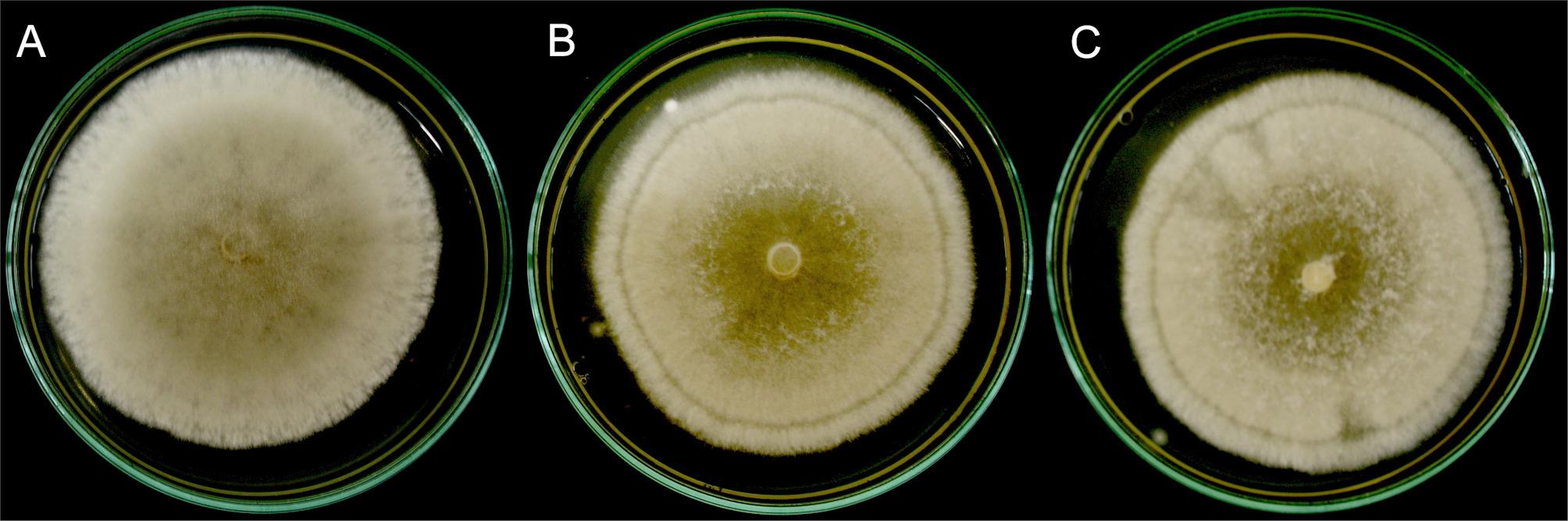
Colony morphology of *B. cinerea* strains on MYA medium. **A** CCg55L wild-type strain. **B** and **C** Transformants CCg55L-A2 and CCg55L-K2, respectively

Having confirmed the presence of viral dsRNA, we evaluated whether CHV1-EP713 expression altered fungal phenotype. Colonies of wild-type *B. cinerea* CCg55L displayed a powdery, brown center and lighter margins (Fig. 5A). In contrast, transformants CCg55L-A2 and CCg55L-K2 showed a feathery morphology with darker centers and more homogeneous light-brown edges, along with a distinct dark-brown concentric ring at the margins (Fig. 5B and C), indicating changes in mycelial growth and pigmentation.

**Fig. 6:**
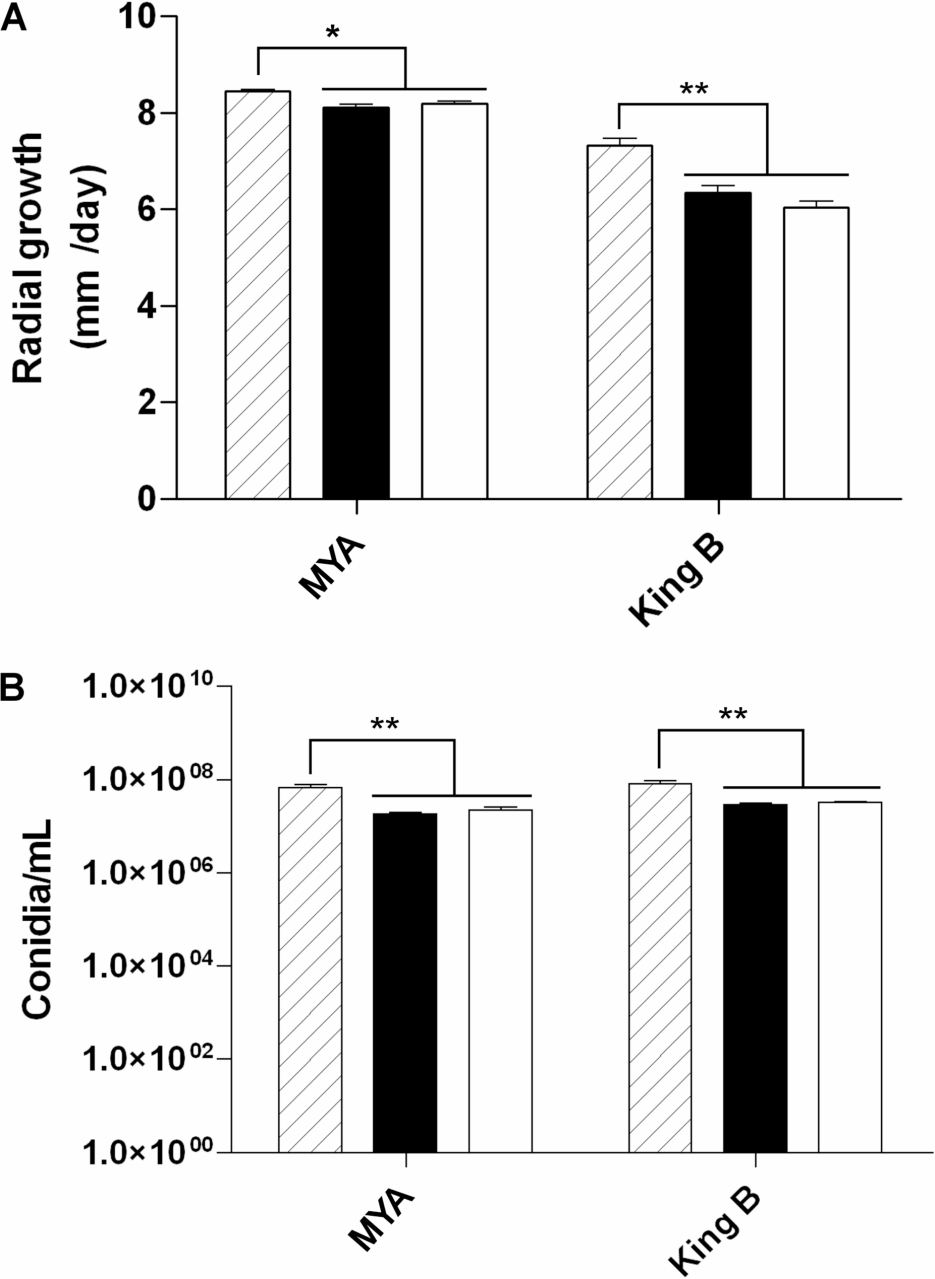
Growth and conidiation rates of *B. cinerea* strains. **A** Radial growth after 4 days in MYA and King B media. **B** Conidiation after 14 days. Bars represent mean ± SE from four biological replicates. One-way ANOVA and Tukey’s test were used for statistical analysis. Horizontal bars group statistically similar data; asterisks denote significance (*p < 0.05; **p < 0.01)

Radial growth and conidiation were quantified on MYA and King B media (Fig. 6A and B). Both transformants exhibited reduced growth rates compared to the wild-type strain, which grew at approximately 7.5–8 mm/day, depending on the culture medium. This rate was 3% and 4% higher than that of the transformants CCg55L-K2 and CCg55L-A2, respectively, when grown on MYA medium. In contrast, on King B medium, although the wild-type strain exhibited slightly slower growth, the differences compared to the transformants were more pronounced, with a growth reduction of approximately 12–15% (Fig. 6A). Conidiation after 14 days was also significantly reduced in CCg55L-A2 and CCg55L-K2, with sporulation levels ranging from 1 × 10⁷ to 5 × 10⁷ conidia/mL, compared to 5 × 10⁷ to 1 × 10⁸ conidia/mL in the wild-type strain CCg55L (Fig. 6B). These differences were statistically significant, indicating that CHV1-EP713 expression affects fungal growth and sporulation under optimal conditions.

### Virulence assays

**Fig. 7:**
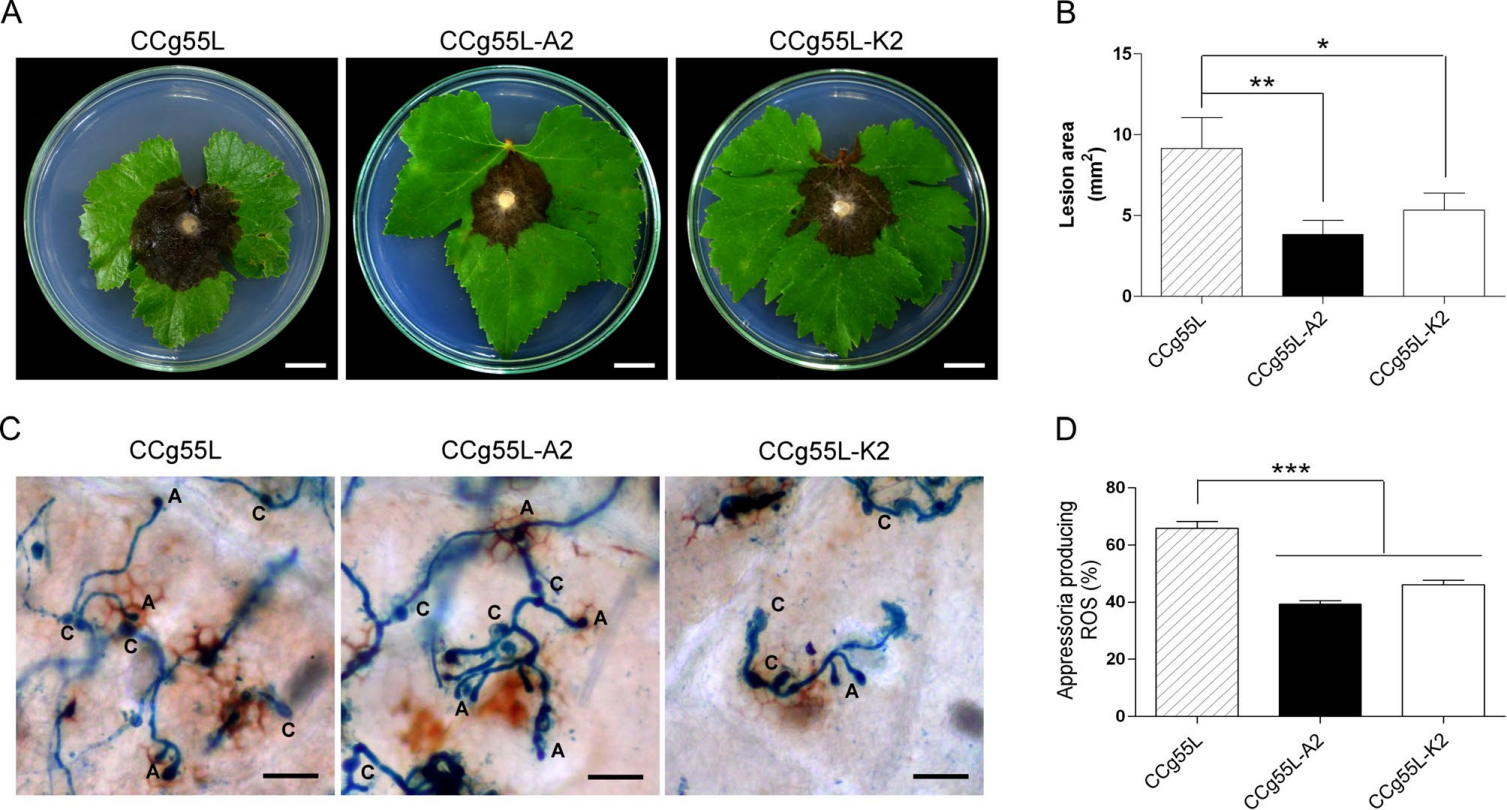
Virulence assays and host oxidative response. **A** Representative images of leaf damage (scale bar = 15 mm). **B** Quantification of lesion area. **C** Oxidative burst detected by DAB and trypan blue staining (scale bar = 60 nm). **D** Number of appressoria associated with ROS production. Data represent the mean ± SE from four biological replicates. Statistical analysis was performed using ANOVA and Tukey’s test. Asterisks indicate significance (*p < 0.05; **p < 0.01; ***p < 0.001)

To assess whether hypovirus expression affects virulence, grapevine leaves were inoculated with actively growing *B. cinerea* mycelia. After five days, lesions caused by the transformants were visibly smaller than those caused by the wild-type strain (Fig. 7A). Quantification confirmed that the wild-type strain caused lesions of ~ 9.15 mm^2^, which were 62% and 46% larger than those produced by CCg55L-A2 and CCg55L-K2, respectively (Fig. 7B). These results suggest that CHV1-EP713 expression reduces the virulence of *B. cinerea*.

### Plant oxidative response to B. cinerea infection

Host oxidative burst in response to *B. cinerea* infection was evaluated using DAB staining to detect hydrogen peroxide production. Leaves were infected with conidia, incubated for 24 h, and stained with DAB and trypan blue. Fewer appressoria-associated oxidative burst sites were observed in leaves infected with the transformed strains compared to the wild-type strain (Fig. 7C). Quantification showed that *B. cinerea* CCg55L induced significantly more ROS-associated appressoria than either transformant, with no significant difference between CCg55L-A2 and CCg55L-K2 (Fig. 7D).

## Discussion

In this work, we expressed the full-length cDNA of the hypovirus CHV1-EP713 in *B. cinerea* through *A. tumefaciens*-mediated transformation and determined that its expression alters the fungal phenotype, resulting in a hypovirulent strain. In this regard, it is important to note that mycoviruses do not exhibit extracellular propagation, and are transmitted either vertically during mitosis or meiosis of the infected fungus, or horizontally via the fusion of compatible hyphae, generally of the same species [3, 14, 40]. For this reason, infection with purified viral particles is limited to spheroplast fusion techniques [25, 41–43]. Other methods to establish mycovirus infection include transfection of virus-free fungal strains with viral mRNA or transformation with a vector that allows viral genome expression [3, 44]. Transformation methods include spheroplast fusion, electroporation, biolistic delivery, and, less frequently, *A. tumefaciens*-mediated transformation [3, 24, 45]. In this study, we used a vector containing the full-length CHV1-EP713 cDNA, carried on an *A. tumefaciens* plasmid, to express the viral genome in *B. cinerea*. Although this method has previously been used to transform *B. cinerea*, allowing the expression of reporter genes such as GFP and β-glucuronidase, as well as selection with hygromycin B [30, 46], it has not been used before to transform the fungus with a vector enabling the expression of a viral genome.

The construction of the p18-XH9 vector, obtained by fusing the pXH9 and p18 plasmids previously digested with the *Sgf*I enzyme, was a key step that enabled the transformation of *B. cinerea* and the expression of the hypovirus genome. Two *B. cinerea* transformants, named CCg55L-A2 and CCg55L-K2, were selected, and CHV1-EP713 expression was confirmed by RT-PCR detection of viral mRNA and by dsRNA purification using CF11-cellulose chromatography. The detection of viral RNAs by RT-PCR indicates that the viral genome was transcribed in *B. cinerea*, and although the GDP promoter and transcription terminator originated from *C. parasitica* [22], these regulatory elements were recognized by the transcriptional machinery of *B. cinerea*. This supports our hypothesis that both fungal species share similar molecular mechanisms. Moreover, the detection of dsRNAs indicates that viral mRNAs were translated in *B. cinerea*, since in hypoviral infections, such RNAs correspond to replicative intermediates and are synthesized exclusively by the viral RNA-dependent RNA polymerase (RdRp) [14]. While both *B. cinerea* and *C. parasitica* belong to the phylum *Ascomycota*, they are classified in different classes: *Leotiomycetes* and *Sordariomycetes*, respectively. Previously, the pXH9 vector had been used to express the CHV1-EP713 genome in *Valsa ceratosperma* and *Phomopsis G-type*, two fungi within the *Diaporthales* order, like *C. parasitica* [24]. Therefore, our results establish *B. cinerea* as the most phylogenetically distant organism in which CHV1-EP713 genome expression has been achieved.

The electrophoretic analysis of dsRNAs from *B. cinerea* CCg55L-A2 and CCg55L-K2, purified by CF11-cellulose chromatography, revealed an approximately 13-kbp band, similar in size to the replicative intermediate of the CHV1-EP713 genome [15]. The presence of this dsRNA band in both transformants, but not in the parental strain *B. cinerea* CCg55L, confirms that the CHV1-EP713 cDNA was not only transcribed but also translated, producing the viral RNA-dependent RNA polymerase. As previously mentioned, the formation of this 13-kbp dsRNA can only occur through the activity of RdRp, which generates replicative intermediates from viral mRNAs [14]. In *C. parasitica*, besides the full-length 13-kbp dsRNA molecule (L-dsRNA), additional molecules between 8 and 10 kbp (M-dsRNAs) and 0.6 to 1.7 kbp (S-dsRNAs) have been identified as defective RNAs derived from the viral genome [47].

Most of the characterized mycoviruses to date are cryptic and/or latent, meaning that infections in host fungi are not associated with any discernible phenotype [48]. However, some mycoviruses are known to reduce virulence or alter fungal development. One such case is CHV1-EP713, which infects the phytopathogenic fungus *C. parasitica* and causes a drastic reduction in virulence against its host, the chestnut tree [12]. Infection with this hypovirus is associated with reduced damage to plant tissue, lower conidiation rates, decreased pigment production, and decreased laccase enzyme activity [12, 14]. These hypovirulence-associated traits have also been observed in fungal strains transformed with the pXH9 plasmid, which enables the expression of CHV1-EP713 cDNA in the recipient fungus [12, 22]. In our study, the first noticeable phenotypic trait in *B. cinerea* transformants CCg55L-A2 and CCg55L-K2 was a change in mycelial morphology when grown on solid media. Compared to the parental strain, which exhibited a powdery mycelium with intense brown coloration at the center and no ring at the periphery, the transformants showed feathery mycelia with a lighter brown center and a distinct dark ring near the edge of the colony. Unlike *C. parasitica*, pigment accumulation in the mycelium of *B. cinerea* has not been well characterized. However, DHN melanin—a pigment responsible for brown coloration—is known to accumulate in *B. cinerea* conidiophores and conidia [49]. The smaller dark areas observed in the transformants suggest a reduction in conidiation, a phenotype similar to that seen in CHV1-EP713-infected *C. parasitica*. This reduction may explain the decreased DHN melanin accumulation in the transformant colonies.

Regarding fungal development, *B. cinerea* exhibited the typical radial mycelial growth pattern, with the colony center representing the oldest region where conidiation initiates. This was observed in both the parental and transformed strains. The transformants also showed slightly slower radial growth compared with the virulent parental strain. Although radial growth differences were relatively small on MYA medium (3–4%), they were more pronounced on King B medium (~ 12–15%). This difference is biologically plausible, as the nutritional composition of King B medium (e.g., glycerol and proteose peptone as the primary sources of C and N, together with phosphate and Mg^2^⁺) differs from that of MYA and can modulate hypovirulence-associated traits by altering the fungal C/N metabolism. Both host gene expression and viral accumulation are known to be sensitive to environmental conditions, which may explain these observations [50–52]. In this context, studies on fungus–hypovirus interactions have consistently shown that virulence attenuation may occur with only slight to moderate effects on in vitro growth, as pathogenicity-related traits are often more sensitive than colony expansion. In our case, this was supported not only by growth measurements but also by altered colony morphology, expression of viral cDNA, and significantly reduced lesion size in planta. Comparable results have been reported for the mycoviruses BcHV1 and LbBV1 in *B. cinerea*, which attenuate virulence and sclerotial production while exerting only mild effects on mycelial growth [53, 54]. Similar phenotypic alterations have also been described in *C. parasitica* infected with CHV1-EP713, as well as in other *B. cinerea* strains carrying hypovirulence-associated viruses. Indeed, infections by hypoviruses, fusariviruses, botybirnaviruses, and mitoviruses are frequently associated with slight to moderate reductions in mycelial growth, whereas the decrease in virulence is typically much more pronounced [54–56].

The bioassays with grapevine leaves provided insights into the virulence of the *B. cinerea* transformants compared to the virulent parental strain. In this type of assay, the fungus deploys its full arsenal of virulence factors to infect plant tissue. This evaluation is particularly relevant, as CHV1-EP713 infection in *C. parasitica* has been linked to a significant reduction in the ability to infect chestnut trees [14]. A qualitative assessment revealed that the transformants CCg55L-A2 and CCg55L-K2 caused less necrotic damage to grapevine leaves than the parental strain CCg55L, with both transformants producing similar lesion sizes. These observations were supported by quantitative analysis and graphical representation of the necrotic areas, which confirmed that although there were no significant differences between the two transformants, both caused significantly smaller lesions than the parental strain.

The mechanism by which the CHV1-EP713 hypovirus reduces virulence in *B. cinerea* remains unknown, although it has been more extensively studied in *C. parasitica*. In that species, hypovirus infection is associated with reduced levels of Gα protein, elevated intracellular cyclic adenosine monophosphate (cAMP), and interference with the MAPK signaling pathway [12]. Notably, CHV1-EP713 downregulates the expression of cpg-1, which encodes the Gα subunit [57]. Deletion of cpg-1 in a virus-free virulent *C. parasitica* strain results in a phenotype similar to that induced by CHV1-EP713, supporting its role in hypovirulence [12, 58]. Although no pathways have yet been identified in *B. cinerea* as targets of CHV1-EP713 or other hypovirulence-inducing viruses, the product of the *bcg1* gene has been characterized as the Gα subunit of the heterotrimeric G protein [59]. Disruption of *bcg1* in *B. cinerea* has been linked to altered colony morphology, slower mycelial growth, and a loss of conidial infectivity, although conidia can still germinate [59]. Interestingly, similar phenotypes were observed in our transformants expressing the CHV1-EP713 genome, including reduced mycelial growth and morphological changes. Although further studies are needed, our findings suggest that CHV1-EP713 expression in *B. cinerea* might affect *bcg1* expression or function. In *C. parasitica*, specific viral proteins are associated with particular phenotypic changes: p29 with reduced sporulation and pigmentation, p40 with decreased sporulation and viral RNA accumulation, and p48 with reduced plant damage and viral RNA accumulation [12, 20, 60].

When *B. cinerea* infects plants, it triggers the production of reactive oxygen species (ROS) in the affected tissues. As a necrotrophic pathogen, *B. cinerea* benefits from host cell death, and the oxidative burst promotes its infection [32]. In our study, qualitative analysis of oxidative responses revealed differences between the parental strain CCg55L and the transformants. After staining with diaminobenzidine (DAB), the brown areas associated with ROS accumulation were smaller in tissues infected by CCg55L-A2 and CCg55L-K2. Additionally, the percentage of appressoria associated with oxidative bursts was significantly lower in both transformants compared to the parental strain.

CHV1-EP713 infection in *C. parasitica* has not been linked to changes in host ROS production. However, in *B. cinerea*, the *bdsod1* gene encodes a superoxide dismutase (SOD-1) enzyme that plays a role in fungal virulence [61, 62]. SODs catalyze the dismutation of superoxide radicals into hydrogen peroxide and oxygen, contributing to ROS detoxification [63]. A *bdsod1*-deficient mutant exhibits reduced growth and virulence in *Arabidopsis thaliana* and *Solanum lycopersicum*, along with decreased H_2_O_2_ accumulation in infected plant tissue [62]. While no direct link has been established between decreased fungal SOD activity and plant H_2_O_2_ levels, it has been reported that SOD-1 secretion is downregulated in hypovirus-infected *C. parasitica* strains [64]. This downregulation of SOD-1 by CHV1-EP713, combined with the known impact of *bdsod1* deletion on *B. cinerea* virulence and ROS accumulation, may help explain the reduced oxidative response and pathogenicity observed in the transformants CCg55L-A2 and CCg55L-K2.

The T-DNA insertion during the transformation of filamentous fungi using *A. tumefaciens* occurs randomly, which means that the T-DNA can integrate into different genomic regions in each transformant [45, 65, 66]. Consequently, analyzing more than one *B. cinerea* transformant helps reduce the likelihood that the observed phenotypic changes are due to disruption of endogenous genes essential for fungal virulence. This is particularly important because *A. tumefaciens*-mediated transformation was initially developed as a tool for random mutagenesis in fungi, similar to its application in plants [45, 65, 66]. As demonstrated by TAIL-PCR in our study, the T-DNA was inserted into a gene of unknown function in CCg55L-A2 and into an intergenic region in CCg55L-K2. Despite these different insertion sites, both transformants displayed the same hypovirulence-associated phenotype. This strongly suggests that the phenotypic changes were not caused by gene disruption due to T-DNA insertion, but rather by the expression of the CHV1-EP713 genome.

The results presented here indicate that the expression of CHV1-EP713 in *B. cinerea* can significantly alter fungal phenotypes, suggesting potential applications in the development of novel biocontrol strategies. Hypoviruses such as CHV1 have long been investigated for their capacity to attenuate fungal virulence—most notably in *C. parasitica*, where CHV1-EP713, a prototypic hypovirus isolated in the 1970s, was shown to markedly reduce both asexual sporulation and pathogenicity. Its successful deployment in the biological control of chestnut blight remains a landmark example of hypovirus-mediated disease management [67]. In their comprehensive review, Xie and Jiang [67] further emphasized the broader promise of hypoviruses, such as SsHADV-1 in *Sclerotinia sclerotiorum*, which not only induce hypovirulence but also overcome barriers, including vegetative incompatibility, thereby broadening the feasibility of field-level mycovirus applications. Building on this foundation, more recent studies have explored hypovirulence-associated mycoviruses across a broader range of phytopathogenic fungi. For example, García-Pedrajas et al. [68] reviewed diverse fungal systems and highlighted both the potential and the practical challenges of mycovirus-based biocontrol, particularly regarding transmission and stability. Similarly, Yu et al. [69] demonstrated that SsHADV-1 can be efficiently transmitted and not only suppresses virulence in *S. sclerotiorum* but also enhances plant growth and resistance. In *B. cinerea*, several hypovirulence-associated viruses have been identified, such as BcHV1, BcFV1, and BcRV1, but their practical use has been constrained by limited horizontal transmission and potential fitness costs [3, 54]. However, the successful heterologous expression of CHV1-EP713 in *B. cinerea*, as demonstrated in this study, may open new avenues for engineering hypovirulence traits into otherwise intractable fungal pathogens. Leveraging well-characterized hypoviruses such as CHV1-EP713 could help overcome some of the limitations observed with native *B. cinerea* mycoviruses. Overall, our findings contribute to the growing evidence that mycoviruses represent promising, sustainable, and environmentally friendly tools for integrated plant disease management.

## Conclusions

The p18-XH9 vector was constructed to enable *A. tumefaciens*–mediated transformation of *B. cinerea*. In the two transformants obtained, the T-DNA was integrated at different genomic loci. Expression of the hypovirus genome in *B. cinerea* led to marked phenotypic changes, including altered mycelial morphology, reduced conidiation, and decreased radial growth. Infectivity assays on grapevine leaves further demonstrated a diminished ability of both transformants to damage host tissue and to trigger ROS production, consistent with a hypovirulent phenotype.

## Supplementary Information

Additional file1 (DOCX 410 kb)

## Data Availability

As requested, the authors will make all data and materials available to other researchers.
