# Supplementary material for: Heterologous expression of the hypovirus CHV1-EP713 full-length cDNA in Botrytis cinerea: transformation with Agrobacterium tumefaciens and evaluation of changes in the fungal phenotype
Source: Biol Res. 2025 Oct 8;58:65. doi: 10.1186/s40659-025-00645-y (PMC12506336; doi:10.1186/s40659-025-00645-y)
Supplement: Supplementary file 1 — Additional file1 (DOCX 410 kb) [file 40659_2025_645_MOESM1_ESM.docx]

**Supplementary material**

**
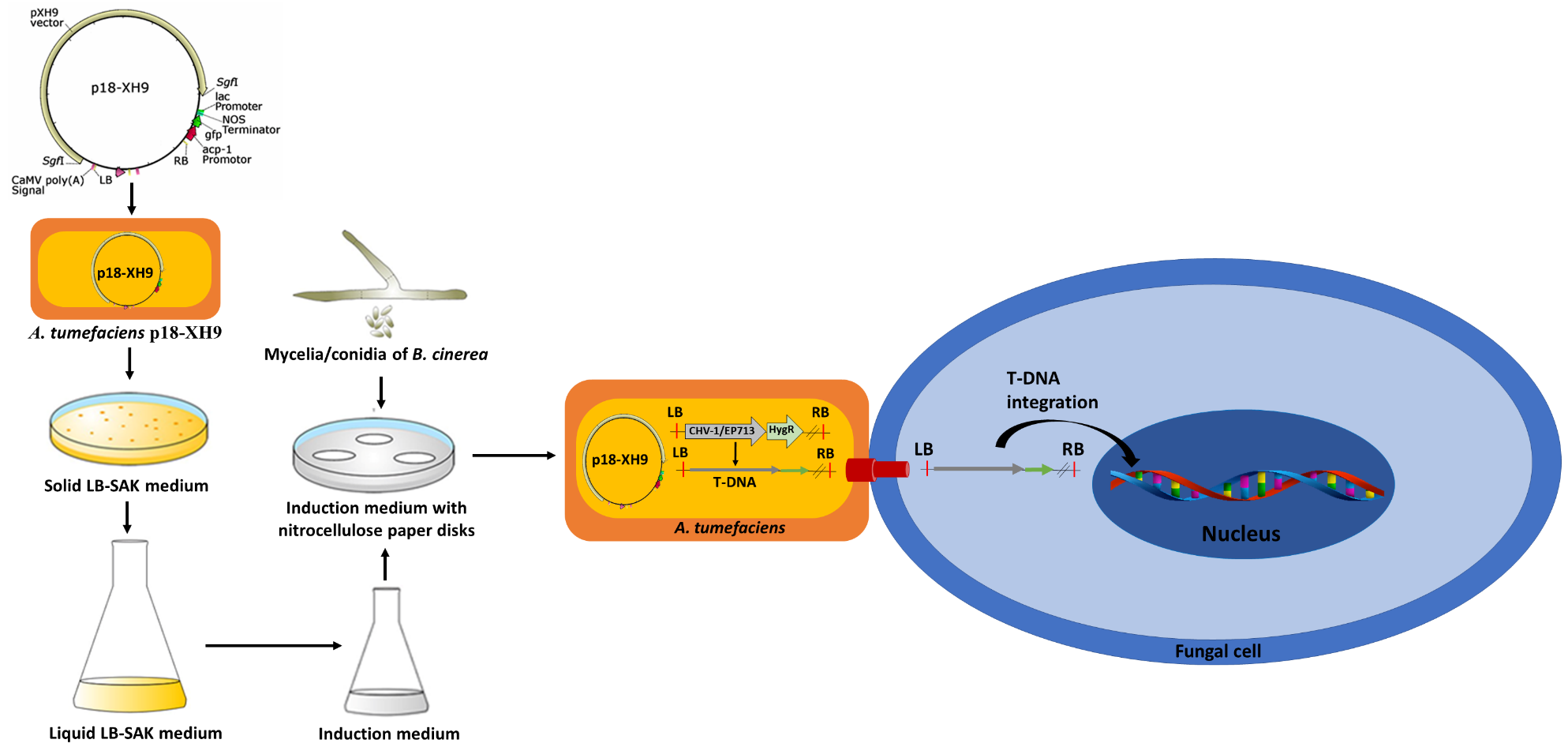
**

**Figure S1**. Schematic representation of the *Agrobacterium tumefaciens*–mediated transformation protocol for *Botrytis cinerea*. A single *A. tumefaciens* colony harboring the vector p18-XH9 was grown for 24 h in liquid medium containing antibiotics. This culture was then diluted into induction medium supplemented with acetosyringone and incubated for approximately 5 h at 28 °C. The bacterial suspension was mixed with *B. cinerea* mycelial or conidial suspensions on the surface of a nitrocellulose filter and co-cultivated for 2 days at 22°C on solid induction medium. Under these conditions, the bacteria induce T-DNA synthesis and activate the transfer system, which delivers the single-stranded DNA (ssDNA) segment to the nucleus of the fungal cell for integration into the fungal genome. Because the T-DNA carries a selectable marker, fungal transformants were subsequently isolated on plates containing medium supplemented with hygromycin B and cefotaxime.

SAK: spectinomycin, ampicillin, and kanamycin.
